# An Uncommon Cause of Acute Transverse Myelitis Following Acinetobacter Baumannii-Associated UTI, Which Responded to Intravenous Pulse Methylprednisolone Alone

**DOI:** 10.7759/cureus.18509

**Published:** 2021-10-05

**Authors:** Dibya J Sharma, Phulen Sarma, Laky Saha, Abunasar M Masroor

**Affiliations:** 1 Internal Medicine: Gastroenterology, Silchar Medical College and Hospital, Silchar, IND; 2 Pharmacology, Postgraduate Institute of Medical Education and Research (PGIMER), Chandigarh, IND; 3 Internal Medicine, Silchar Medical College and Hospital, Silchar, IND; 4 Cardiology, Silchar Medical College and Hospital, Silchar, IND

**Keywords:** acute transverse myelitis, acinetobacter, uti, pulse, methyl prednisolone, neurological, urinay tract, autonomic, motor, sensory

## Abstract

Acute transverse myelitis (ATM) is a non-compressive localized inflammation involving one or more levels of the spinal cord due to various etiologies characterized by motor weakness, sensory impairments, and autonomic dysfunction. It can be idiopathic or primary or secondary due to infection, autoimmune disorder, connective tissue disorder, and uncommonly after vaccination which came to the limelight during the ongoing massive vaccine drive against coronavirus disease 2019 (COVID-19).

We report a case of a 21-years-old male who presented with gradually progressive weakness of both lower limbs following urinary tract infection (UTI) with a history of similar illness in the family which improved with high dose methylprednisolone and antibiotic therapy followed by physical rehabilitation. A diagnosis of long segment ATM possibly following UTI was suggested after ruling out other secondary causes and was confirmed by magnetic resonance imaging (MRI) of the spinal cord. Asymmetric symptoms and signs with small lesions involving <two vertebral segments, peripheral lesion, presence of Lhermitte’s sign and relapsing-remitting course distinguish ATM from more debilitating disorder multiple sclerosis (MS) in patients with family history. Infection like UTI can precipitate ATM as well as UTI may develop along with neurogenic lower urinary tract dysfunction (NLUTD) even after recovery from the motor and sensory impairment. Patients with acute transverse myelitis need to be on regular follow up particularly those with subacute presentation and positive family history to rule out relapse and development of multiple sclerosis. Common etiologies like UTI may precipitate uncommon disorders like ATM.

## Introduction

Acute transverse myelitis (ATM) is an inflammatory disorder of the myelin sheath of the spinal cord which can precipitate acutely within hours to days or can start insidiously and progress over one to four weeks [1]. It can be idiopathic (15- 34%) or contributed by antecedent secondary causes including connective tissue disorder (20.5%), infarction of spinal cord (18%), multiple sclerosis (10-62%), infection-related (17%), neuromyelitis optica (1-17%) and uncommonly post-vaccination [2,3]. Acute presentation with extensive spinal cord involvement, backache, delayed presentation to hospital, spinal shock, progression to syringomyelia, electromyography (EMG) showing denervation, presence of protein 14-3-3 in cerebrospinal fluid (CSF) is associated with the worse outcome as compared to the subacute presentation which has a good prognosis [2-4]. While one-third of patients make a good recovery with no residual defect, another one-third have partial recovery, and the remaining 30% of patients either die or do not recover [5]. Post-vaccination ATM has been reported after taking polio vaccine (OPV), Japanese B encephalitis vaccination, vaccination for measles-mumps and rubella (MMR), hepatitis B vaccine, diphtheria, pertussis, tetanus toxoid vaccination, rabies vaccine, influenza vaccine as well as post-COVID vaccination, and the time from vaccination to onset of ATM is variable (ranges from five days to three months) in most cases (up to nine years reported) [6].

The index case that we reported was admitted with acute transverse myelitis with a history of similar weakness in his mother a few years back which was associated with urinary tract infectionand other possible secondary causes were ruled out.

## Case presentation

The index case was a 21-years-old male without any addiction or allergy history who presented with weakness in both lower limbs for five days and arthralgia of both knee joints which started insidiously. The weakness progressed over the next three days to cause inability to walk which was preceded by fever with chill and rigor for two days prior to the development of weakness*.* After two days he also developed weakness in both upper limbs with difficulty in buttoning, combing hair, lifting the arm above the shoulder, truncal weakness, and inability to keep sitting posture and had to roll in bed to change position. Motor weakness was not associated with any sensory impairment, cranial nerve involvement, restricted eye movement or eye pain or loss of consciousness, or seizure. However, there was acute retention of urine with burning micturition and diminished filling sensation of the bladder for which he was catheterized. The patient also gave a history of constipation. No past history of similar weakness, trauma, intravenous drug abuse, jaundice due to viral hepatitis, diabetes, thyroid disorder, genital ulceration, tuberculosis, unsafe sexual behavior, joint pain, oral ulceration, Reynaud’s phenomenon, hair loss, skin rash, urticaria, chronic diarrhea, lymphadenopathy, parotid swelling, vesicles in the distribution of dermatome were present in the patient. A family history of similar weakness was present in his mother; however, there was no family history of diabetes, tuberculosis, hepatitis B or C infection, thyroid disorder, autoimmune disorder, connective tissue disorder including sarcoidosis was reported.

On general clinical examination, the patient had a body mass index (BMI) of 23 kg/m^2^ with the normal respiratory rate (16/min), sinus tachycardia (102/min), elevated temperature (38 °C), and normal blood pressure (110/70 mm Hg) without any other significant finding. Neurological examination revealed increased tone in lower limbs more in the extensor group of muscles, reduced power in both lower (3/5) and upper limbs (4/5), exaggerated jerks in both upper and lower limbs, loss of crude touch, and vibration sense below T7 vertebral level, extensor plantar reflex bilaterally and decreased anal tone with absent anal reflex suggestive of upper motor neuron lesion with autonomic dysfunction. Cerebellar function, higher mental function, cranial nerves, skull, and spine examination were unremarkable without any evidence of neck stiffness, Kernig’s sign, and Lhermitte’s sign.

**Figure 1:**
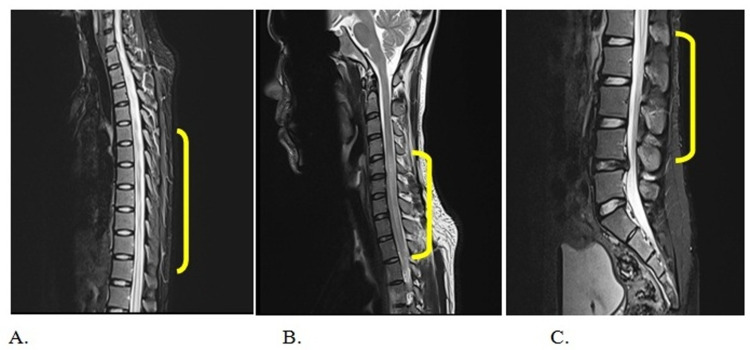
Sagittal T2 sequences of (A) Thoracic spine, (B) Cervical spine, and (C) Lumbar spine demonstrating long segment hyperintensity and variable cord swelling with minimal post-contrast enhancement and no significant canal stenosis

**Figure 2:**
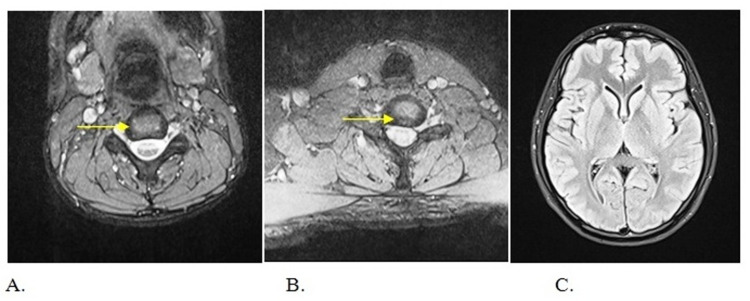
Sequence axial slices demonstrating central signal hyperintensity Sequence axial slices at the level of (A) Cervical spine, (B) Thoracic spine demonstrating central signal hyperintensity (yellow arrow) (C) MRI Brain was normal.

The patient was hospitalized for further evaluation and management. Laboratory evaluation revealed lymphopenia with normal complete blood count, elevated ESR (40 mm/hr), normal C-reactive protein (CRP), D dimer, serum ferritin, serum lactate dehydrogenase (LDH), blood sugar, thyroid function test, VDRL (Venereal Disease Research Laboratory) test, Montoux’s test, autoimmune profile like antinuclear antibody (ANA), viral markers for hepatitis A, E, B, C, human immunodeficiency virus (HIV), SARS-CoV-2 antigen test, scrub serology, parasite test for malaria (Pf/Pv test), chest X-ray were normal. Urine examination revealed 10-15 pus cells/HPF (high power field) and RBCs, which on culture demonstrated growth of *Acinetobacter* *baumannii* which was sensitive to ciprofloxacin, piperacillin and tazobactam, and imipenem/cilastatin. Ophthalmological examination was unremarkable for optic neuritis and neuromyelitis optica (NMO). MRI scan of the brain including orbit was unremarkable whereas MRI of the spine showed hyperintensity involving more than two-thirds of the cross-sectional area extending up to multiple (more than four) vertebral levels at cervical, thoracolumbar spine with minimal post-contrast enhancement and variable enlargement of cord suggestive of acute transverse myelitis as shown in Figures 1, 2. At lumbar L4 - L5 level, asymmetric diffuse disc bulge, central protrusion, and anterior thecal sac indentation were noted. Cerebrospinal fluid (CSF) analysis showed increased protein (130 mg/dL), normal glucose (50 mg/dL), slightly elevated cell count (10 cells/mm^3^*^ ^) *with 80% polymorphonuclear cell (PMN) and normal adenosine deaminase (ADA) level. CSF oligoclonal band was not done due to the non-availability of the facility at our center.

Treatment

Our patient received injection methylprednisolone, 1 gm daily, intravenously in 500 ml normal saline for five days followed by a tapered dose of oral methylprednisolone for two weeks along with I.V. ciprofloxacin, pregabalin, multivitamin, calcium supplementation, and physical rehabilitation.

Follow up

The patients’ motor symptoms improved gradually over one week of starting treatment and became fully ambulatory on day 10. His sensory signs also resolved and the indwelling bladder catheter could be removed on day six of starting treatment after which the patient was discharged from hospital on the 12th day of admission with advice to continue regular physiotherapy. On follow-up after one month, the patient was doing well without any residual *disability*.

## Discussion

Idiopathic ATM was diagnosed as per the 2002 Consortium Working Group consensus on transverse myelitis and both included inclusion and exclusion criteria listed in Table 1 [1].

**Table 1:** Criteria for diagnosis of idiopathic acute transverse myelitis

CRITERIA OF INCLUSION	CRITERIA OF EXCLUSION
Motor, sensory, and dysfunction of the autonomic system due to spinal cord pathology	Exposure to past spinal radiation within 10 years
Bilateral symmetric or asymmetric involvement	Neurological deficit consistent with anterior spinal artery thrombosis
Sensory level of spinal cord	Arterio-venous malformation of spinal cord
Exclusion of external cord compression on imaging (MRI)	Clinical or serological proof of connective tissue disorder
Spinal cord inflammation revealed by CSF pleocytosis, increased IgG index, or enhancement with gadolinium on imaging	Infection-related ATM due to syphilis, Lyme disease, Mycoplasma, Herpes simplex, Zoster, EBV, CMV, Enterovirus, HIV, etc.
Symptoms reaching a peak between 4 hr to 21 days from onset or awakening	No evidence of Multiple sclerosis on brain MRI
	No evidence of optic neuritis on Ophthalmological and clinical evaluation

Acute myelitis is preceded by prodromal symptoms in case of viral infection similar to MS and NMO although in most cases viral etiology remains undetermined. Post-infectious ATM is suspected based on clinical indicators like fever, meningismus, rash, concurrent systemic infection, recurrent genital ulceration, lymphadenopathy, and presence of the immunocompromised state. CSF analysis reveals neutrophilic pleocytosis with elevated protein count. ATM may be completely characterized by long segment (more than three) and central cord involvement whereas incomplete ATM has a short segment (less than three) and peripheral spinal cord involvement. Complete ATM has a low risk of recurrence and progression to MS although patients with anti-Sjo¨gren’s and NMO immunoglobulin (Ig)G antibodies are prone to relapse. Incomplete ATM is more susceptible for recurrence and development of MS, particularly when associated with brain lesions and oligoclonal bands in CSF [7]. The etiology of transverse myelitis is variable and ranges from infection to autoimmune disease and is listed in Table 2 [8].

**Table 2:** Etiological profile of acute transverse myelitis

ETIOLOGY OF TRANSVERSE MYELITIS
Multiple sclerosis, NMO, Acute disseminated encephalomyelitis (ADEM), Systemic autoimmune disorder- SLE, Behcet disease, Mixed connective disease, Systemic sclerosis, Antiphospholipid syndrome, Celiac disease, etc.
Infection associated ATM (Viral): Measles, Paramyxovirus, herpes virus, EBV, CMV, Influenza, Hepatitis virus (A, E, B, C), HIV, dengue, Japanese encephalitis, Parvovirus B19, Cytomegalovirus, Enterovirus 71, Human coronavirus (SARS-CoV-2) etc. Infection associated ATM (Bacterial): Mycoplasma, Chlamydia psittaci, Chlamydia pneumoniae, Acinetobacter baumannii, Scrub typhus, Salmonella paratyphi, Tuberculosis, Syphilis, Leptospira, Brucellosis etc.
Infection associated ATM (Fungal): Aspergillus, actinomyces, coccidiodes, Cryptococcus etc. Parasite: Schistoma spp., Toxocara spp., Toxoplasma gondii, Taenia solium, Acanthomoeba spp., Paragonimus spp. etc Paraneoplastic syndromes: Anti Ri antibody, Anti- amphiphysin antibody, Anti GAD65 antibody, NMDAR antibody
Myelitis associated with atopy, Drugs and toxins, TNFα inhibitors

*Acinetobacter* spp. is associated with UTI, pneumonia, or nosocomial infection after neurosurgical and invasive procedures (mechanical ventilation, thoracic drainage, arterial, venous or intrathecal catheterization). It is a gram-negative coccobacillus that is sensitive to treatment with fluoroquinolone, carbapenem antibiotics although multidrug resistance has been documented. *Acinetobacter baumannii* infecting catheter of intrathecal pump leading to acute transverse myelitis has been documented [9]. Acute transverse myelitis is diagnosed on the basis of clinical, laboratory, and specific MRI findings of the spinal cord and it has a variable extent of involvement as well as outcome depending on the etiology as depicted in Table 3 [10].

**Table 3:** Comparison of outcome depending on etiology of ATM and MRI findings ATM=Acute transverse myelitis; MRI=Magnetic resonance imaging

ETIOLOGY	DIAGNOSIS	OUTCOME
Multiple Sclerosis	MRI: Lesion confined to the posterior and peripheral area of the spinal cord, CSF: reveals oligoclonal bands	The outcome is good, recurrence rate 50%
Systemic disease (connective tissue disorder)	Severe motor and autonomic disorder MRI: large, centromedullary lesion in SLE CSF: > 30 cells	Outcome poor
Infection-related myelopathy	Extensive motor and autonomic involvement MRI: Extensive medullary lesion often involving the central portion of cervical and dorsal cord CSF: Presence of >30 cells without oligoclonal bands Serology unconfirmed in most	Prognosis relatively better
Radiation myelopathy	Radiation exposure in past (lag period variable up to one decade) MRI: Cord showing elevated signal intensity with localized swelling that atrophies progressively CSF: Parameters within normal limit	Good prognosis in early presentation (up to 4 months); Poor in delayed presentation

High-dose intravenous methylprednisolone (MPS) constitutes the cornerstone of therapy and it is associated with rapid recovery, decreased disability with few adverse events if administered in the acute phase. The postulated mechanism of action of MPS includes inhibition of macrophage function, lymphocyte proliferation as well as modulation of tumor necrosis factor (TNF), interleukin (IL)-1, IL-2 activity. Other treatment options include intravenous cyclophosphamide for patients with complete ATM and plasma exchange for complete ATM with motor symptoms has been found to be beneficial [4]. Our patient has completely recovered without any residual disability with the use of high-dose intravenous methylprednisolone alone along with intravenous fluoroquinolone antibiotic therapy.

## Conclusions

Although viral infection is the most common etiological factor for infection associated ATM; bacterial, fungal, and parasitic infection can also precipitate it. Bladder dysfunction is the most common disability reported post acute transverse myelitis and hence urine examination is prerequisite during evaluation of ATM at the onset as well as after recovery from the disease. ATM patients with positive family history need to be regularly followed up to monitor for relapse and development of MS. In such cases role of prophylactic therapy with disease modifying drugs to prevent relapse and progression to MS need to be ascertained. Our case report has highlighted that there may be an association of UTI due to *Acinetobacter baumannii* with acute transverse myelitis .
